# A battery dataset for electric vertical takeoff and landing aircraft

**DOI:** 10.1038/s41597-023-02180-5

**Published:** 2023-06-02

**Authors:** Alexander Bills, Shashank Sripad, Leif Fredericks, Matthew Guttenberg, Devin Charles, Evan Frank, Venkatasubramanian Viswanathan

**Affiliations:** 1 Department of Mechanical Engineering, Carnegie Mellon University, Pittsburgh, PA USA; 2 Airbus Aˆ3, Santa Clara, CA USA

**Keywords:** Aerospace engineering, Batteries

## Abstract

Electric vertical takeoff and landing aircraft have a unique duty cycle characterized by high discharge currents at the beginning and end of the mission (corresponding to takeoff and landing of the aircraft) and a moderate power requirement between them with no rest periods during the mission. Here, we generated a dataset of battery duty profiles for an electric vertical takeoff and landing aircraft using a cell typical for that application. The dataset features 22 cells, comprising a total of 21,392 charge and discharge cycles. 3 of the cells use the baseline cycle while each of the other cells vary either charge current, discharge power, discharge duration, ambient cooling conditions, or end of charge voltage. While it was designed to mimic the expected duty cycle of an electric aircraft, this dataset is relevant for training machine learning models on battery life, fitting physical or empirical models for battery performance and/or degradation, and countless other applications.

## Background & Summary

Accurate models of battery performance and degradation are critical for the development of safe and efficient electric aircraft^1^. These models require validation data from cells operating at representative conditions. While cycling datasets exist for many cell chemistries and use profiles^2^, there is a lack of accessible datasets for cells operating under the unique duty cycles of electric vertical takeoff and landing (EVTOL) aircraft. Such datasets are crucial for developing safe and reliable battery technology for EVTOL aircraft and for informing regulations governing the manufacturing and use of EVTOL aircraft^1^. We previously used this dataset to develop a machine learning model of battery performance and degradation^3^. The landing segment of an EVTOL aircraft duty cycle, where the battery must sustain a high power for ~100 seconds, is particularly challenging for the battery because the cell is already in a depleted state with reduced performance. In contrast, fixed-wing aircraft need high power at takeoff, but require much lower power for landing^4–6^. Here, we generated an experimental battery performance dataset consisting of 21,392 cycles across 22 cells specific to the power requirements of an EVTOL aircraft.

In contrast to the application-specific protocol developed here, most previous datasets developed generic cycling protocols. Over the last decade, several private and public research institutions, universities, and laboratories^7–16^ have published battery cycling and degradation datasets mainly focused on generic cycling protocols considering varying discharge and charge currents, thermal conditions, and cell materials^2^. Since lithium-ion battery degradation can vary depending on use profile, cell chemistry, and cell-to-cell variation, many diverse datasets are needed to adequately sample degradation paths^15,17^. Alongside, there have been several efforts to standardize and publish more battery data to enable further development of machine learning and physical degradation models^18–20^, however, standardization does not fully alleviate the need for application-specific datasets.

To help mitigate the path dependence of battery degradation, engineers and scientists have developed datasets intended to be representative of the intended use cases of batteries. For example, automotive battery testing is performed using testing profiles called ‘drive cycles’ such as the Federal Urban Driving Schedule, and its European and Asian equivalents^21–23^. Aerospace-related battery datasets have thus far been confined to fixed wing^24^ or satellite datasets^25^. Further, there is a lack of test profiles that are considered representative of aerospace operating conditions.

Given the lack of openly accessible datasets that follow the characteristic EVTOL aircraft duty cycle, we generated a nominal EVTOL aircraft mission cycle and subjected commercially available cells to it. The resulting dataset will provide a set of baseline duty cycles to compare with future EVTOL battery data and to inform regulations governing use of batteries in EVTOL aircraft. Each mission profile is based on the following format:

- **Take-off:** the cell is discharged at a high constant power
- **Cruise:** the cell is discharged at a lower constant power for a longer duration
- **Landing:** the cell is discharged at high constant power for a slightly longer period of time than takeoff
- **Rest:** the cell is allowed to rest until it has cooled to a temperature of less than 27 °C or for at least 15 minutes
- **Charging:** the cell is charged using a constant current-constant voltage (CC-CV) charging protocol
- **Rest:** The cell is allowed to rest until cell temperature reaches 35 °C, then allowed to rest 15 minutes further before beginning the next cycle.

**Fig. 1:**
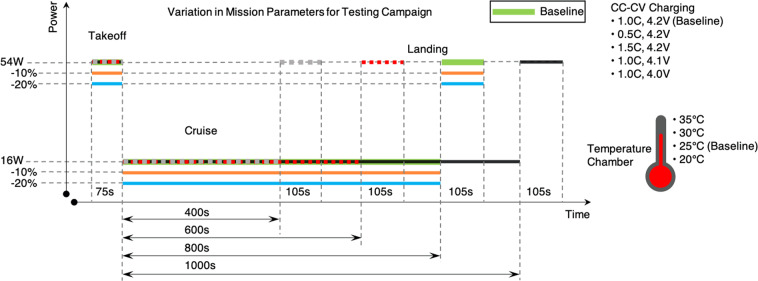
Depiction of EVTOL duty cycle, showing the baseline and variations in the parameters.

A depiction of the canonical EVTOL profile is shown in Fig. 1. During each test, temperature, voltage, and current along with discharge capacity and charge capacity are recorded. The profile is parameterized to test different conditions which could be encountered on a mission, varying the discharge power during flight, charge current, charge voltage, ambient temperature, and cruise length, each varied in a one-at-a-time fashion.

**Fig. 2:**
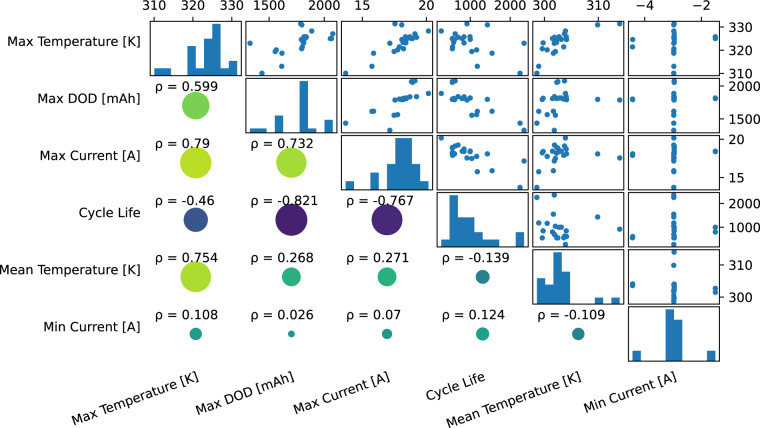
Correlations between the cycling parameters. The best predictor of cycle life is depth of discharge, with a Pearson correlation coefficient of −0.821, followed by the maximum current with a Pearson correlation coefficient of −0.767. Maximum temperature also has a moderate correlation with cycle life, but charging current (minimum current) and mean temperature have a relatively small effect.

The ‘longest-lived’ cell was VAH12, with a cycle life of 2,348 cycles. This cell had a power reduction of 20% for takeoff, landing, and cruise, with all other variables remaining at the baseline. VAH12 had a duty cycle featuring a low depth of discharge, low temperatures, and low currents. The ‘shortest-lived’ cell was VAH07 with a cycle life of 275 cycles. This cell had an upper cutoff charge voltage of 4.0 V (lower than the standard 4.2 V cutoff). Other associations between cycling variables are shown in Fig. 2.

## Methods

This work utilized Sony-Murata 18650 VTC-6 cells. This cell has a rated capacity of 3000mAh at a nominal voltage of 3.6 V. The manufacturer-specified maximum continuous discharge rate is 10 C with an 80 °C upper temperature cut-off. This cell is appropriate for evaluation in EVTOL applications as it can sustain high power demand while providing a cell specific energy of 230 Wh/kg.

**Fig. 3:**
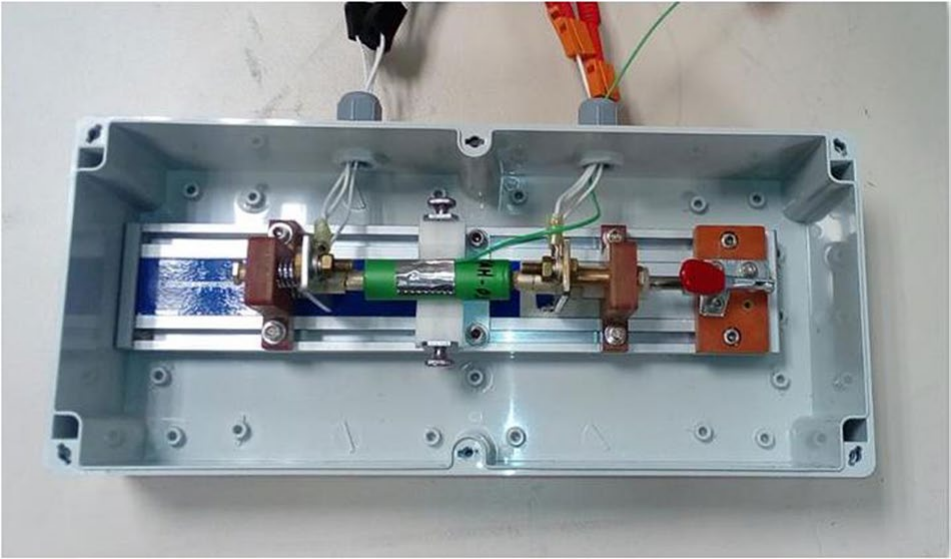
Photograph of cell testing setup.

All cells were tested in an Arbin 200 A cylindrical cell holder paired with a BioLogic BCS-815 modular battery cycler. Figure 3 shows the cell in the testing enclosure. Cells with a specified temperature were placed in a temperature chamber that was maintained at that temperature. The cell can surface temperatures were measured via a thermocouple fixed to the cell body with aluminum tape.

Generation of the EVTOL data set was developed from the baseline power profile for a nominal EVTOL flight given in Table 1 and a series of variations, each of which modified a single variable in the baseline mission profile. The test descriptions are given in Table 2. For each mission profile, cells were cycled until cell voltage reached 2.5 V or cell temperature reached 70 °C during discharge. To measure cell energy capacity a reference performance test (RPT) was performed at the start of each aging test campaign and following each subsequent set of 50 mission cycles. Each RPT consisted of measuring capacity by discharging the cell at a discharge rate of C/5 from 4.2 V until the voltage dropped below 2.5 V alongside an internal resistance measurement at 20% and 60% depth of discharge. After all discharge cycles a rest period was observed to allow the cell to cool to 30 °C. Once 30 °C cell temperature was reached a CC-CV charge profile was started with constant current charge at 1 C, followed by constant voltage charge until charge current decayed to C/30 to a (nominally) 4.2 V end of charge voltage.

**Table 1:** Baseline Mission Parameters (Charge).

Phase	Definition	End Criteria
Take-off	54 W	t = 75 s
Cruise	16 W	t = 800 s
Landing	54 W	t = 105 s
Rest 1	0 A	*T* < 27 °C
CC Charge	1 C	*V* > 4.2
CV Charge	V = 4.2	*I* < *C*/30
Rest 2	I = 0	*T* < 35 °C

**Table 2:** Descriptions of the Testing and Validation dataset.

Cells	
Mission Profile	Description
VAH01	Baseline mission
VAH02	Cruise time, *t*_*c*_=1000 s
VAH05	Power reduction of 10 percent for takeoff, cruise, and landing
VAH06	CC-CV charge cycle at C/2 CC – C/30 CV
VAH07	End of charge voltage at 4.0 V
VAH09	Thermal chamber temperature of 20 °C
VAH10	Thermal chamber temperature of 30 °C
VAH11	Power reduction of 20% for takeoff, cruise, and landing
VAH12	Cruise time, *t*_*c*_=400 s
VAH13	Cruise time, *t*_*c*_=600 s
VAH15	Cruise time, *t*_*c*_=1000 s
VAH16	CC-CV charge cycle at 1.5 C CC – C/30 CV
VAH17	Baseline mission
VAH20	CC-CV charge cycle at 1.5 C CC – C/30 CV
VAH22	Cruise time of 1000 s
VAH23	End of charge voltage at 4.1 V
VAH24	CC-CV charge cycle at C/2 CC – C/30 CV
VAH25	Thermal chamber temperature of 20 C
VAH26	Cruise time of 600 s
VAH27	Baseline
VAH28	Power reduction of 10 percent for takeoff, cruise, and landing
VAH30	Thermal chamber temperature of 35 °C

## Data Records

The raw cell testing data was collected in multiple files over the lifetime of the cell. These files were then concatenated for each cell, and the published cell data contains these concatenated files. The files are saved in comma-separated values (CSV) format files, with the following headings:

- **time_s** Time since beginning of experiment in seconds
- **Ecell_V** Terminal voltage
- **I_mA** Cell current in milliamperes; positive represents charge, and negative represents discharge
- **EnergyCharge_W_h** The amount of energy supplied to the cell during charge in watt-hours
- **QCharge_mA_h** The amount of charge supplied to the cell during discharge in watt-hours
- **EnergyDischarge_W_h** The amount of energy extracted from the cell during discharge in watt-hours
- **QDischarge_mA_h** The amount of charge extracted from the cell during discharge in milliampere-hours
- **Temperature__C** The cell surface temperature as measured by a thermocouple attached to the cell casing in degrees Celsius
- **cycleNumber** Cycle number as recorded by the cell tester
- **Ns** Cycle segment

Additionally, we have calculated the internal resistance for each cell and saved it in a separate set of files in the same repository. The raw cell files are saved as VAH00.csv, while the impedance files are saved as VAH00_impedance.csv (where 00 is replaced by the cell’s serial number). The headings of the impedance files are:

- **20%_1_second** Impedance at 20% DOD measured after 1 second of low current
- **20%_30_second** Impedance at 20% DOD measured after 30 seconds of low current
- **60%_1_second** Impedance at 60% DOD measured after 1 second of low current
- **60%_30_second** Impedance at 60% DOD measured after 30 seconds of low current
- **cycle_numbers** Cycle number

The data are available under a CC-BY-4.0 license at 10.1184/R1/14226830.v2^26^.

## Technical Validation

### Validation of degradation in cell capacity and voltage

**Fig. 4:**
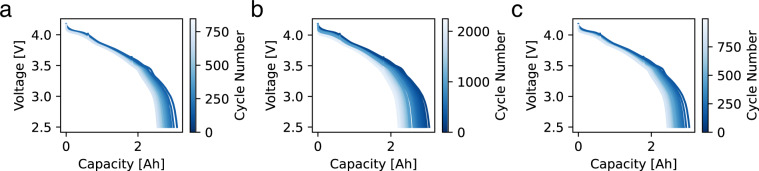
Voltage vs capacity as a function of cycle number for 3 cells in the dataset.

**Fig. 5:**
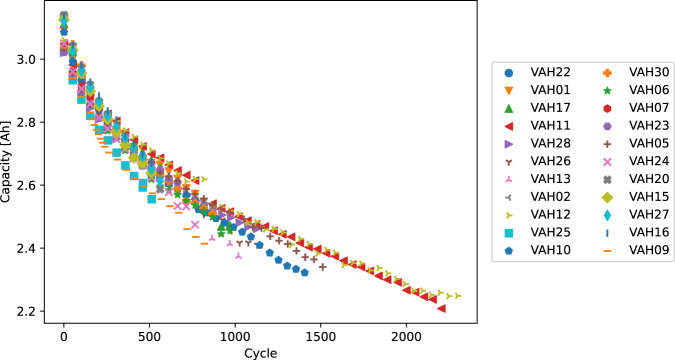
Capacity vs cycle life for all cells as measured in the RPTs.

**Fig. 6:**
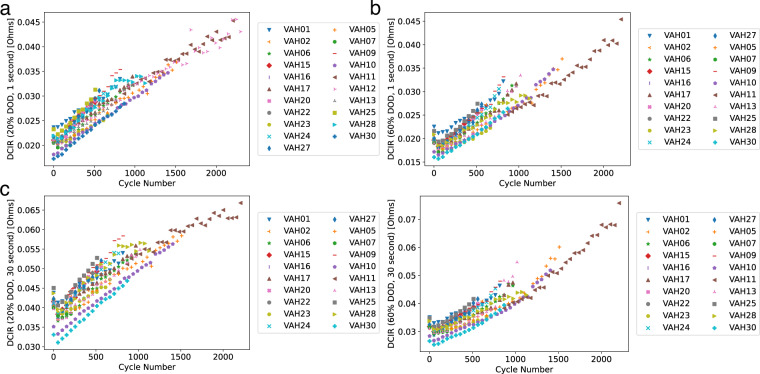
DC Internal Resistance (DCIR) for each cell at all RPT’s measured at 1 second and 30 seconds after slowing the current at 20% and 60% depth of discharge.

Figure 4 shows voltage vs capacity for the reference performance tests for each cell over its life. At the beginning of its life, the cell has a nominal capacity of 3 Ah. As expected, the capacity declines over life, leading to a contraction of the curve along the capacity axis. Figure 6 shows DC internal resistance (DCIR) vs cycle life for each cell over its life. All cells show increase in resistance; and for most cells, the increase is linear or slightly superlinear. The resistance was measured by allowing the cell to relax for a period of 30 seconds at a current of C/50 during the reference performance tests at depths of discharge of 20% and 60%. DCIR can be calculated by measuring the voltage before the current is lowered and after, then dividing the difference in voltage by the difference in current. Figure 5 shows the capacity of each cell vs cycle life, as measured in the RPTs. The capacity follows an expected pattern, starting at 3 Ah and declining with a decelerating rate of decline. We note that as a result of the defined end criteria (V < 2.5 V or T > 70 °C), different cells have different capacities at the end of life, in contrast to typical aging studies in which the end criteria is based on the capacity.

**Fig. 7:**
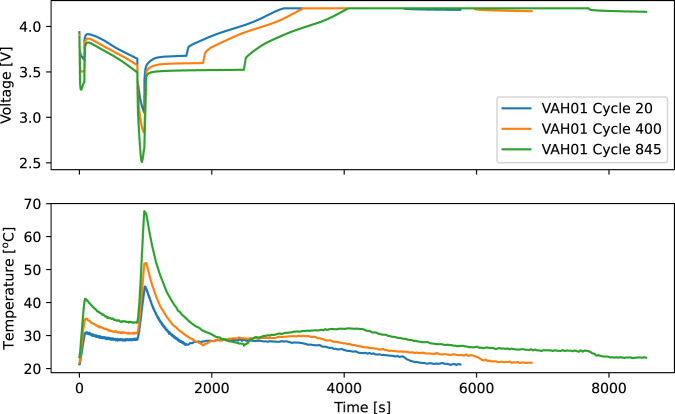
Voltage and temperature vs time for a cycle at the beginning, middle,and end of life. Temperature rises and voltage falls as the battery ages and capacity increases and internal resistance decreases.

Figure 7a shows voltage vs time for 3 typical mission cycles, while 7b shows temperature vs time for the same 3 cycles, one from the beginning of life, one from the middle of life, and one from the end of life. These demonstrate the effects of dropping capacity and increasing internal resistance: as the capacity and resistance decrease, the current must rise to meet the power demands of an EVTOL aircraft; consequently, the temperature rises due to ohmic heating.

### Validation of data processing and other known issues

A very low fraction of cycles in the data showed evidence of tester malfunction or loss of power to the testing facilities^26^ The following is a list of known issues in the data:

- **VAH05 cycle 1000** Missing a Capacity Test after cycle 1000 and the rest period from the preceding cycle
- **VAH09 cycle 64** Apparent tester malfunction; cell did not charge all the way to 4.2 V
- **VAH09 cycle 92** Apparent tester malfunction; discontinuity during cycle
- **VAH09 cycle 154** Capacity test stopped early
- **VAH09 cycle 691** Discontinuity during the cycle
- **VAH10 cycle 249** Data stops during mission discharge cycle
- **VAH10 cycle 631** Data stops during mission discharge cycle, followed by a long break
- **VAH10 cycle 735** Data collection stops, then resumes after a period of rest
- **VAH10 cycle 1151** Data collection stops, then resumes after a period of rest
- **VAH11 cycle 817** Data stops during capacity test
- **VAH11 cycle 1898** Missing data
- **VAH13 cycles 816 & 817** Missing capacity test
- **VAH25 cycles 461 & 462** Extra capacity test
- **VAH26 cycles 872 & 873** Data stopped and started during the rest period following a mission cycle
- **VAH27 cycle 20** Unphysical voltage spike
- **VAH27 cycle 585** Longer than nominal rest period
- **VAH28 cycles 256 & 257** Extra capacity test
- **VAH28 cycles 619 & 620** Apparent tester malfunction; data stopped and started during the CV charging segment
- **VAH28 cycles 1066 & 1067** Apparent tester malfunction; data stopped and started during the CV charging segment

## Data Availability

No custom code was used in the generation of this dataset. The data were analyzed using the Julia programming language and plotted using matplotlib^27,28^.
